# Compound Characterization and Metabolic Profile Elucidation after In Vitro Gastrointestinal and Hepatic Biotransformation of an *Herniaria hirsuta* Extract Using Unbiased Dynamic Metabolomic Data Analysis

**DOI:** 10.3390/metabo10030111

**Published:** 2020-03-16

**Authors:** Laura Peeters, Anastasia Van der Auwera, Charlie Beirnaert, Sebastiaan Bijttebier, Kris Laukens, Luc Pieters, Nina Hermans, Kenn Foubert

**Affiliations:** 1 Natural Products & Food Research and Analysis (NatuRA), Department of Pharmaceutical Sciences, University of Antwerp, Universiteitsplein 1, 2610 Antwerp, Belgium; anastasia.vanderauwera@uantwerpen.be (A.V.d.A.); sebastiaan.bijttebier@gmail.com (S.B.); nina.hermans@uantwerpen.be (N.H.); kenn.foubert@uantwerpen.be (K.F.); 2 Adrem Data Lab, Department of Mathematics—Computer Sciences, University of Antwerp, Middelheimlaan 1, 2020 Antwerp, Belgium; charlie.beirnaert@uantwerpen.be (C.B.); kris.laukens@uantwerpen.be (K.L.)

**Keywords:** *Herniaria hirsuta*, Caryophyllaceae, saponins, urolithiasis, machine learning, dynamic metabolomics

## Abstract

*Herniaria hirsuta* L. (Caryophyllaceae) is used for treatment of urinary stones and as a diuretic. Little is known about the active compounds and the mechanism of action. The phytochemical composition of *H. hirsuta* was comprehensively characterized using UHPLC-UV-HRMS (Ultrahigh-Performance Liquid Chromatography-Ultraviolet-High Resolution Mass Spectrometry) data. An in vitro gastrointestinal model was used to simulate biotransformation, which allowed the monitoring of the relative abundances of individual compounds over time. To analyze the longitudinal multiclass LC–MS data, XCMS, a platform that enables online metabolomics data processing and interpretation, and EDGE, a statistical method for time series data, were used to extract significant differential profiles from the raw data. An interactive Shiny app in R was used to rate the quality of the resulting features. These ratings were used to train a random forest model. The most abundant aglycone after gastrointestinal biotransformation was subjected to hepatic biotransformation using human S9 fractions. A diversity of compounds was detected, mainly saponins and flavonoids. Besides the known saponins, 15 new saponins were tentatively identified as glycosides of medicagenic acid, acetylated medicagenic acid and zanhic acid. It is suggested that metabolites of phytochemicals present in *H. hirsuta*, most likely saponins, are responsible for the pharmaceutical effects. It was observed that the relative abundance of saponin aglycones increased, indicating loss of sugar moieties during colonic biotransformation, with medicagenic acid as the most abundant aglycone. Hepatic biotransformation of this aglycone resulted in different metabolites formed by phase I and II reactions.

## 1. Introduction

Urinary stone disease is considered as an important healthcare problem that affects 10–15% of the population in the developed world, but the incidence can be as high as 20–25% in the Middle East, with a peak at ages 20 to 40 years. In addition, the disease is characterized by its high recurrence rate, about 50% in 10 years and 75% in 20 years [1,2,3,4,5,6].

Over the years, many remedies and surgical treatments have been described, varying from dietary recommendations to interventional procedures. Endoscopic management, both ureteroscopic and percutaneous, offers an efficient and efficacious way to treat stones [6]. Nowadays, extracorporeal shock wave lithotripsy (ESWL) is widely used for stone fragmentation without invasive instrumentation of the body. However, the aforementioned therapies show some significant side effects such as residual stone fragments that can act as a nidus for new stone formation [5,7]. Existing oral treatments with evidence that supports their long-term efficacy to prevent kidney stones only include a handful of drugs. Thiazide diuretics, potassium citrate and allopurinol have been used as therapy for more than 30 years. However, their adverse effects prevent long term consumption [2,8]. 

Phytotherapy might be useful as an alternative treatment. Some herbal remedies have been used for centuries and have been shown to be effective against urolithiasis, although the mechanism of action is often not well established through systematic pharmacological and clinical studies. Many plants have been and are still used to treat urinary stones, such as *Phyllanthus niruri*, *Zea mays*, *Agropyron repens* and *Herniaria hirsuta* [5,9]. 

An aqueous extract of the aerial parts of *H. hirsuta* (hairy rupturewort) is an herbal medicine widely used against urolithiasis and which contains diuretic properties. The Arabic name, herras lehjer, is translated as stonebreaker and refers to its use as a traditional herbal remedy for urinary stones. The beneficial effects of the extract have been demonstrated in several studies [9,10,11,12,13,14]. Even though a lot of research has been done to prove the activity of *H. hirsuta* against urolithiasis, little is known about the active compounds and the exact mechanism of action. Previous phytochemical research on *Herniaria* species revealed the presence of saponins, flavonoids and coumarins [15,16,17,18]. Four herniariasaponins have been reported in *H. hirsuta*: herniariasaponins E, F and G, three monodesmosidic derivatives of medicagenic acid and herniariasaponin H, a bidesmosidic derivative of medicagenic acid [17,19]. Literature suggests that the antilithiatic potential of *H. hirsuta* is attributed to saponins or metabolites thereof [17,20,21]. 

It is well known that many natural products are so-called prodrugs, which become active after biotransformation [22]. Nevertheless, this aspect is usually overlooked when searching for new therapeutic agents using classical approaches. After oral administration, an herbal extract is inevitably brought into contact with gastrointestinal enzymes and intestinal microflora, which might lead to biotransformation of the compounds. Saponins are high-molecular-weight glycosides, containing a triterpenoid or steroidal sapogenin aglycone covalently linked to one or two sugar chains via an ether or ester glycosidic bond, referred to as monodesmosidic or bidesmosidic saponins, respectively. Before absorption in the gastrointestinal tract, saponins are presumably hydrolyzed with loss of sugar chains [23]. The aglycones or metabolites thereof may be absorbed and may further be biotransformed in the liver to the ultimate active components [24]. In this study, the biotransformation of a well-characterized *H. hirsuta* extract was investigated in an in vitro gastrointestinal model followed by hepatic biotransformation. The longitudinal multiclass data were subjected to different data analysis workflows to screen and tentatively identify metabolites formed after in vitro biotransformation.

## 2. Results and Discussion

### 2.1. Identification of Compounds

**Figure 1:**
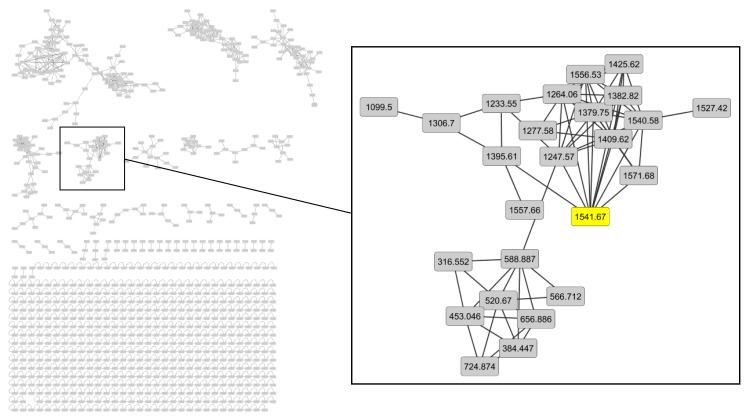
Molecular network in negative ion mode with the cluster containing herniariasaponin H shown in detail.

**Figure 2:**
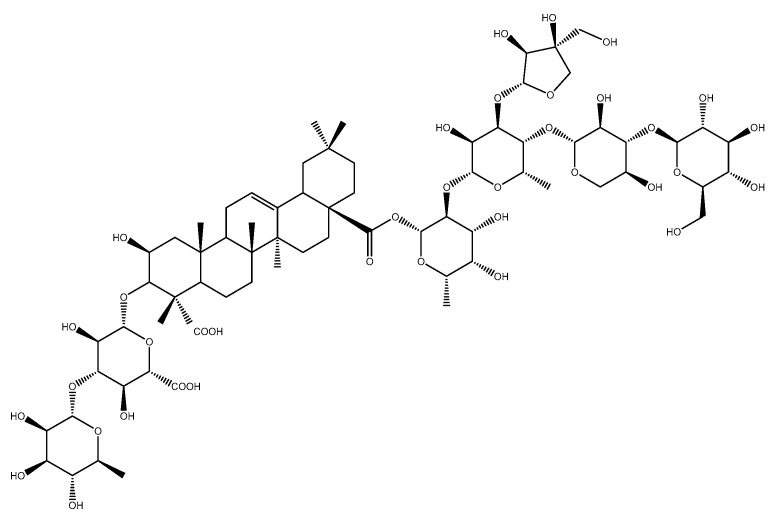
Chemical structure of herniariasaponin H.

A dereplication workflow performed on the extract of *H. hirsuta* resulted in a molecular network containing different clusters, composed of different nodes connected by edges which define the degree of similarity between the MS/MS spectra. Compounds that are structurally related are linked to each other. Mass differences between the nodes were studied and provided information about hitherto unidentified compounds. Major clusters were analyzed to tentatively identify unknown compounds. As an example, the cluster containing herniariasaponin H is shown in Figure 1. Herniariasaponin H (Figure 2) is the most abundant saponin previously observed in *H. hirsuta* and served as starting point for identification of other compounds in this cluster. 

Structures were assigned to peaks only when both the mass/charge (*m*/*z*) ratios and the molecular formulae of the precursor ion and product ions were in agreement. Additional information about the proposed structures was often provided by photodiode array (PDA) spectra and retention times. However, the information was not always sufficient for full elucidation of the structural composition at an acceptable confidence level. Additional information was obtained from published data.

All amMS and PDA data used for peak identification are listed in Table 1. The table also specifies the literature consulted to obtain additional information in order to confirm compound identity. In accordance with previous research, mainly flavonoids and saponins were identified [15,16,17,18,19,20,21]. However, many of the identified compounds have not been reported previously in *H. hirsuta*.

Flavonoids were mainly observed as glycoconjugates, i.e., a flavonoid aglycone attached to one or more sugar moieties. Several flavonoid compounds were identified using analytical standards. Others were tentatively identified with ddMS^2^. Collision-induced fragmentation resulted in product ions caused by elimination of sugar moieties, indicating glycosidic *O*-linkages [24]. Among others, rutin, narcissin and quercetin-3-*O*-(2″-*O*-α-L-rhamnopyranosyl)-β-D-glucuronopyranoside were identified, in agreement with previous studies [17,20]. However, isorhamnetin-3-robinobioside and isorhamnetin-3-[3-feruloylrhamnosyl-(1→6)-galactoside], previously reported to be present in *H. fontanesii*, were not detected in *H. hirsuta*, which is probably due to variances in phytochemical profile between different plant species [18].

A rich diversity of saponins was detected, differing in the presence of various aglycones, a different composition of sugar chains and varied linkages of sugar moieties. Fragmentation led to the distinct presentation of product ions caused by elimination of sugar moieties, indicating glycosidic *O*-linkages. The product ions in the MS^2^ spectra provide additional structural information about the sugar residues and the aglycones of the fragmented saponins. Losses of *m*/*z* 132, 146, 162 and 176 were observed, corresponding to pentose, deoxyhexose, hexose and uronic acid moieties, respectively. Three different aglycone moieties were observed with fragment ions at *m*/*z* 543.33234, 541.31787 and 501.32074, corresponding to acetylated medicagenic acid, zanhic acid and medicagenic acid, respectively [25,26]. 

Several other compounds, such as free phenolic and hydroxycinnamic acids, were detected. Amongst others, caffeic, *p*-coumaric and chlorogenic acids were identified using analytical standards. The coumarins herniarin and umbelliferone, previously reported to be present in *Herniaria* species, were not detected. This variation in compounds is probably due to differences in origin or regional climate [17,20]. More than one chromatographic peak was often present for the same precursor ion, indicating the presence of structural isomers with different linkage positions and/or different sugar moieties.

**Figure 3:**
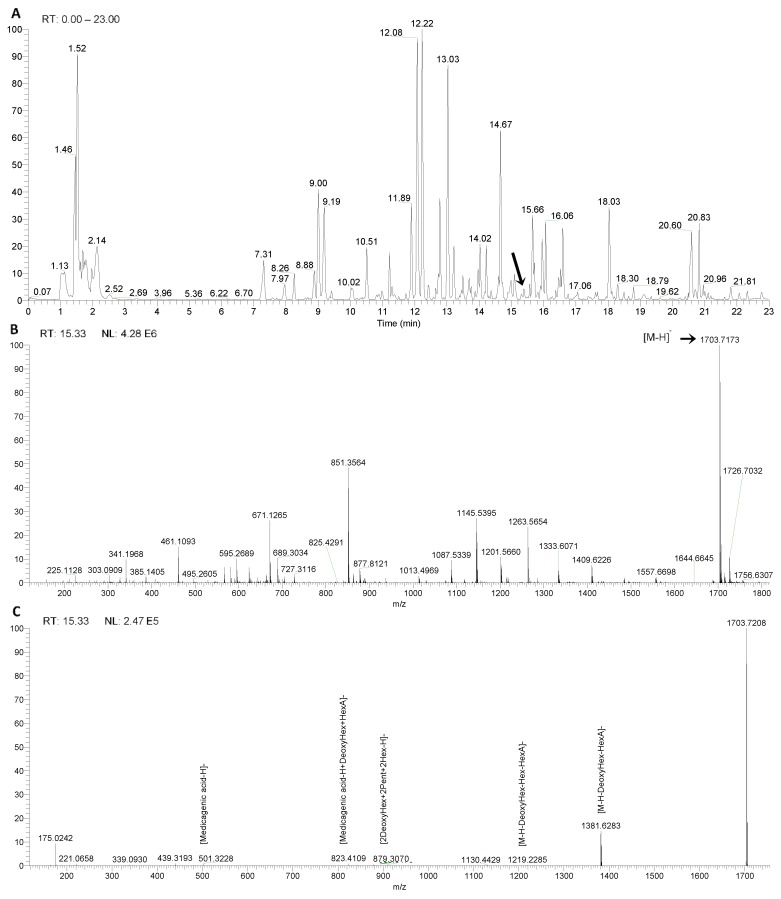
LC–MS data for an unknown saponin. (**A**) Chromatogram obtained for the extract of *Herniaria hirsuta*. (**B**) MS scan of peak at 15.33 min in the heated electrospray ionization (HESI) negative mode. (**C**) MS^2^ fragmentation spectrum of the ion at *m*/*z* 1703.71734.

Figure 3 illustrates the identification of a hitherto unknown saponin. As shown in Figure 3A, the unknown saponin has a retention time of 15.33 min. Figure 3B shows a molecular ion at *m*/*z* 1703.71838 [M-H]^−^. Since saponins contain the elements C, H and O, 19 molecular formulas are possible within an error range of 5 ppm.

The sugar residues and the aglycone were identified from the MS^2^ spectra as shown in Figure 3C and Table 2. The product ion of *m*/*z* 1381.62830 [M-H-176-146]^−^ results from the loss of uronic acid (176 u) and deoxyhexose (146 u). 

The ion at *m*/*z* 879.30701 originates from a sugar chain containing two deoxyhexosyl (146 u), two pentosyl (132 u) and two hexosyl (162 u) units. The ion at *m*/*z* 823.41089 [aglycone-H+146+176]^−^ represents the aglycone attached to a deoxyhexose unit (146 u) and a uronic acid moiety (176 u). The ion at *m*/*z* 501.32275 is characteristic for medicagenic acid as aglycone, resulting from the loss of sugar residues. The ion at *m*/*z* 439.31934 [aglycone-H-18-44]^−^ results from an additional loss of water (18 u) and CO_2_ (44 u). This suggests a bidesmosidic saponin with R_1_ comprising uronic acid and deoxyhexose and R_2_ comprising two pentosyl, two deoxyhexosyl and two hexosyl moieties, with a hexosyl moiety in a terminal position. Combining all the information with the retention time and literature data, the saponin with *m*/*z* 1703.71734 supports a molecular formula of C_76_H_120_O_42_ and was tentatively identified as medicagenic acid attached to three deoxyhexosyl, two hexosyl, two pentosyl and a uronic acid moiety [15,16,17,18,19,20,21]. However, MS^n^ does not provide enough information for absolute structural characterization. Nevertheless, the use of a hybrid orbitrap mass analyzer enabled the tentative identification of 15 saponins that have not been reported before in *H. hirsuta* (indicated with ^e^ in Table 2). Full structural characterization is not the main goal in this project, as the complete herbal extract is subjected to biotransformation studies.

### 2.2. Gastrointestinal Biotransformation

Herbal extracts comprise a mixture of compounds, covering a wide range of bioactive constituents, aside from active compounds or prodrugs. As the main compounds are classified as flavonoids and saponins, extensive biotransformation after oral intake can be expected. Therefore, gastrointestinal biotransformation of the lyophilized extract was simulated in vitro to monitor the levels of the identified compounds using amMS and UV data. However, gastrointestinal enzymes and fecal microflora present in the samples cause a lot of matrix interference, increasing the complexity of the data. The multiclass samples are measured as a function of time, adding a longitudinal aspect to the complex data and impeding the interpretation of the data. The concentration of precursor compounds and metabolites can increase, decrease or show any combination of these patterns during biotransformation. 

**Figure 4:**
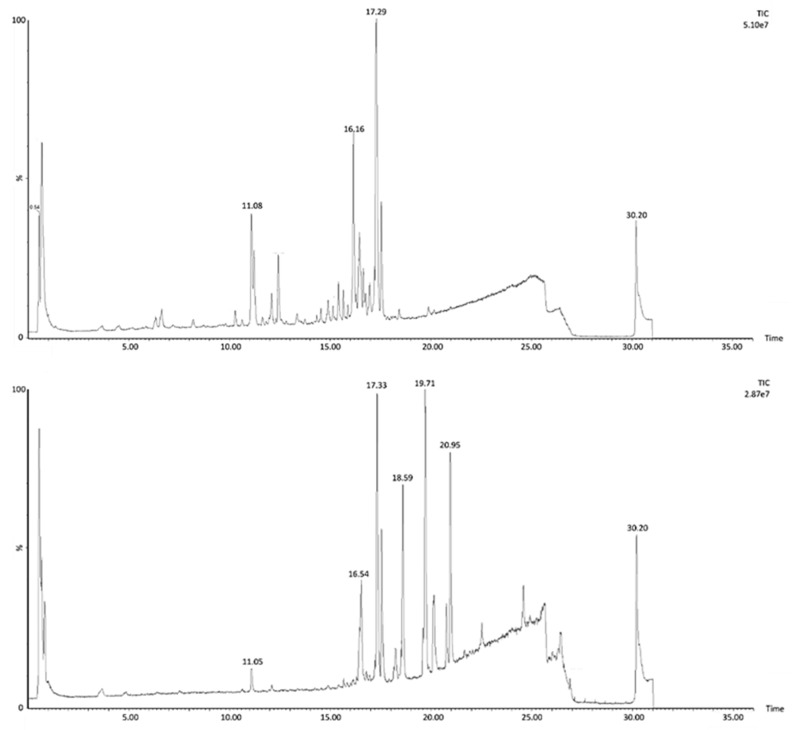
Total ion chromatogram in negative ion mode of *H. hirsuta* before and after biotransformation.

Figure 4 shows the chromatogram of the *H. hirsuta* extract before and after biotransformation. Before biotransformation (t_0_), peaks are mostly attributed to the tentatively identified compounds. After the colonic phase of gastrointestinal biotransformation, the chromatogram contains peaks of compounds, metabolites and matrix interferences, originating from enzymes and fecal microflora, which are absent at t_0_. 

Processing the chromatograms of every time point for every compound allows monitoring of the intensity over time. However, this manual approach is time-consuming and will only provide information about the abundance of previously identified compounds. Manual screening for metabolites is comparable to looking for a needle in a haystack because of the complexity of the chromatograms and matrix interferences.

**Figure 5:**
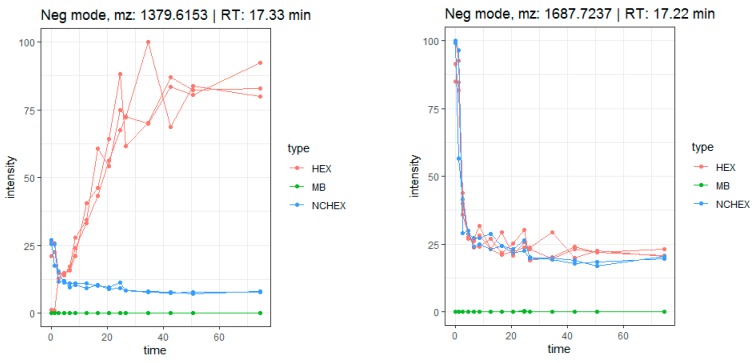
Time profile of the biotransformation of compounds **46** (left) and **45** (right) in samples (HEX), negative controls (NCHEX) and blank (MB).

Generally, for most tentatively identified compounds, a decrease in relative abundance is observed mainly at the colonic phase, caused by bacterial degradation, suggesting formation of metabolites. However, some compounds, mainly saponins, show an increase in relative abundance over time. It is suggested that this increase is induced by conversion of one compound into another, caused by loss of one or more sugar moieties. For example, compound **46** (medicagenic acid + three deoxyhexosyl + two pentosyl + one glucuronyl moiety) shows an increase in relative abundance and reaches a maximum after 24 h colon phase, which is not observed in the negative control samples (NCHEX) (Figure 5). In addition, the signal was absent in the blank (MB), as expected. At the same time, a decrease of compound **45** (medicagenic acid + one hexosyl + four deoxyhexosyl + two pentosyl + one glucuronyl moiety) is observed. The increase of compound **46** can be related to the decrease of compound **45**, caused by the loss of a hexosyl and a deoxyhexosyl moiety. The decrease is observed in both samples and negative control during the stomach phase and the small intestinal phase. At these time points, there is no difference in samples and negative control as the negative controls only lack fecal bacteria during the colonic phase. Since identification based on MS-data is not absolute, only tentative conclusions can be drawn from these observations. However, the data support the theory that gastrointestinal biotransformation leads to a loss of sugar moieties in saponins.

An automated data analysis workflow, previously described by Peeters et al. and Beirnaert et al., was used to screen for metabolites [23,27]. The workflow resulted in 11,072 features in negative ion mode, of which 4846 were annotated as isotopes. In positive mode, 5242 features were detected after exclusion of isotopes. 

**Figure 6:**
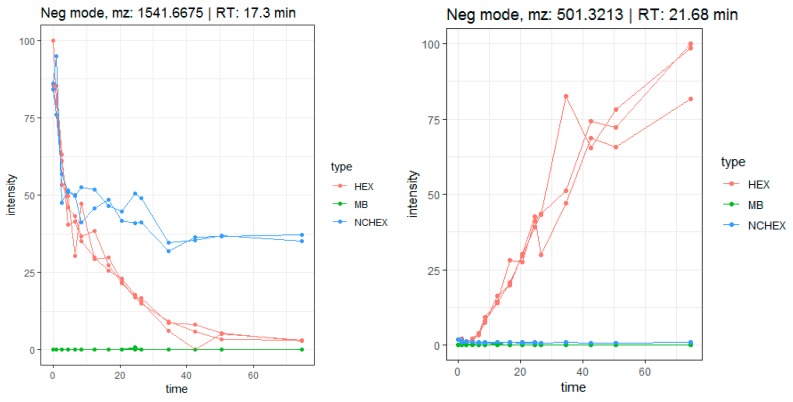
Time profile of gastrointestinal biotransformation of herniariasaponin H (*m*/*z* 1541.6675 [M-H]^−^) and medicagenic acid (*m*/*z* 501.3213 [M-H]^−^).

**Figure 7:**
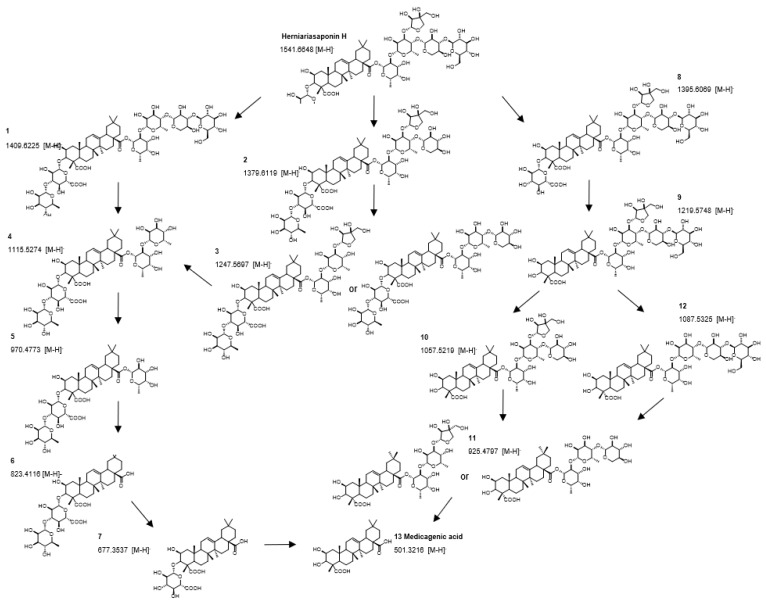
Metabolic pathway of herniariasaponin H by in vitro gastrointestinal biotransformation.

Firstly, the metabolic pathway of herniariasaponin H (*m*/*z* 1541.6675, rt: 17.3 min) was studied in detail to verify if the workflow was suitable for herbal extracts. The time profile of herniariasaponin H shows a decrease in intensity over time, especially during the colonic phase, which is not observed in the negative control samples (NCHEX) (Figure 6). In addition, the signal is absent in the method blank (MB). The feature is rated as notable based on the results of tinderesting, the interactive Shiny app developed in R, with a score of 1 and a ranking of 242 out of 6226 features. During biotransformation, stepwise elimination of sugar moieties is observed resulting in formation of the aglycone (Figure 7). All intermediate metabolites show a tinderesting score of at least 0.98, meaning that they are all rated as interesting. Finally, the aglycone medicagenic acid (*m*/*z* 501.3213; rt: 21.68) is formed, showing an increase in signal over time in the samples, with a tinderesting score of 0.998 (Figure 6).

Features with a tinderesting score higher than 0.8 were selected for in-depth exploration with the following inclusion criteria: a reasonable intensity with good-quality MS/MS spectra and a retention time between 2 and 25 min.

The main challenge in the analysis of a large amount of analytical data remains in the identification and annotation of the features of interest. However, the data analysis workflow ranks features according to one single score taking the longitudinal aspect and the three sample classes into account, which facilitates selection of features of interest. On the other hand, determination of the molecular formula of compounds with a molecular mass above 1000 Dalton becomes more challenging. Therefore, this step in the data analysis process is still performed manually. 

**Figure 8:**
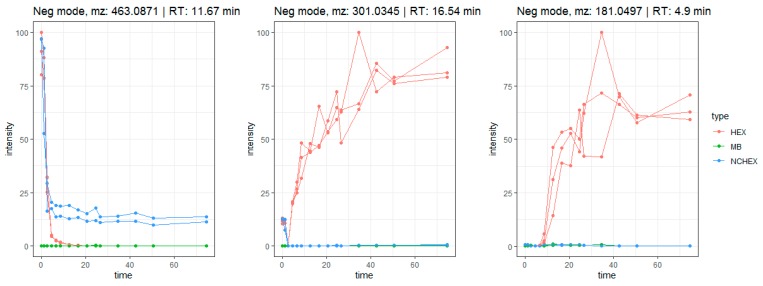
Time profiles of biotransformation of isoquercetin (*m*/*z* 463.0871 [M-H]^−^), quercetin (*m*/*z* 301.0345 [M-H]^−^) and a metabolite (*m*/*z* 181.0497 [M-H]^−^) tentatively identified as 3,4-dihydroxyphenylpropionic acid or 3-methoxy-4-hydroxyphenylacetic acid.

As expected, the features included in the in-depth exploration could be attributed to saponins, flavonoids and their metabolites. Saponins (compounds **35**–**41**, **43**–**45**, **47**–**48**, **51**, **58**, **59** and **61**) show the same biotransformation pathway as the studied example, herniariasaponin H. In brief, the intensity of the signal decreases over time due to stepwise elimination of sugar moieties, leading to formation of the aglycone. The observed biotransformation of flavonoids is similar. In nature, flavonoids mainly occur as glycosides. Gastrointestinal biotransformation causes deglycosylation, leading to formation of the aglycone. For example, quercetin glycosides are abundantly and ubiquitously present flavonoids in nature. The aglycone is formed after biotransformation of several precursors, such as isoquercetin and rutin. Isoquercetin (*m*/*z* 463.0871; rt: 11.67 min) has a tinderesting score of 0.86 and shows a decrease in intensity over time (Figure 8). The decrease is already observed in the stomach phase and continues in the small intestinal phase and colonic phases until the signal is completely absent. In the negative control samples, the signal stays stable during the colonic phase, because there are no bacteria present. The signal is absent in the method blank. For quercetin (*m*/*z* 301.0345; rt: 16.54 min), an increase in intensity is observed over time, which is not observed in the negative control or method blank (Figure 8). The feature is rated as interesting with a tinderesting score of 1. This suggests deglycosylation of isoquercetin leading to the formation of quercetin. 

**Figure 9:**
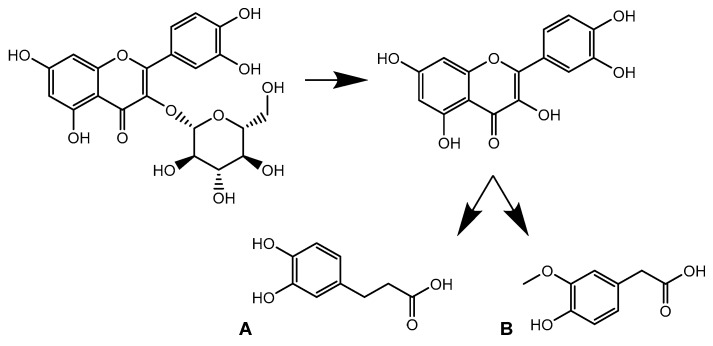
Biotransformation pathway of isoquercetin showing quercetin as intermediate metabolite which is further transformed into 3,4-dihydroxyphenylpropionic acid (**A**) or 3-methoxy-4-hydroxyphenylacetic acid (**B**).

A feature with *m*/*z* 181.0497 and rt: 4.9 min was included in the in-depth exploration as it showed a tinderesting score of 0.992. The feature shows an increase in intensity over time, mainly during the colonic phase (Figure 8). The metabolite was tentatively identified as 3,4-dihydroxyphenylpropionic acid (A) or 3-methoxy-4-hydroxyphenylacetic acid (B). Both compounds were previously described as metabolites of quercetin [28,29]. Based on the obtained spectra, absolute structural characterization is not possible. Nonetheless, the product ion could be identified as a metabolite of quercetin. This shows that despite the increase in intensity of quercetin, the compound is further biotransformed (Figure 9). 

### 2.3. Liver Biotransformation

**Figure 10:**
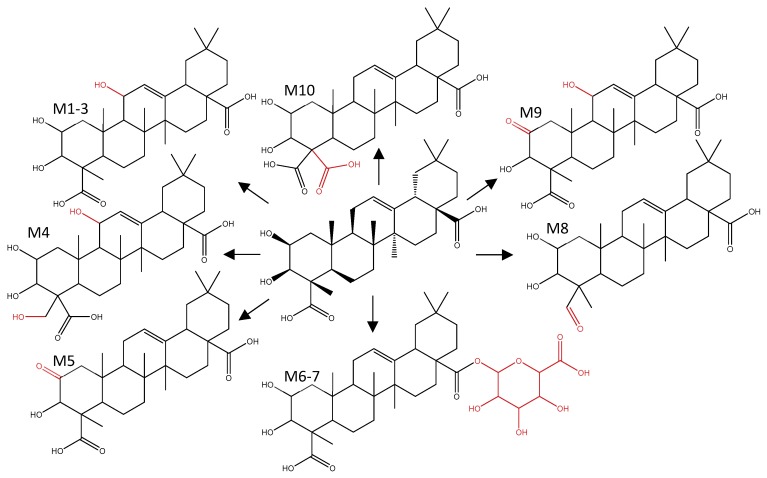
B Metabolic pathway of medicagenic acid after in vitro hepatic biotransformation.

Figure 10 shows an overview of the proposed metabolic pathway of medicagenic acid after S9 biotransformation. For metabolites **1**–**3** (**M1**–**3**), three different chromatographically separated peaks were observed (rt 17.39; 17.60 and 18.45 min), suggesting the presence of three different structural isomers. This was confirmed by the prediction provided by Meteor. The software proposed hydroxylation at C-11, C-24, or C-29. Double hydroxylation (**M4**), oxidation of a hydroxy group (**M5**) and a combination of hydroxylation and oxidation (**M9**–**10**) were also predicted by Meteor. The nontarget screening approach confirmed the formation of the predicted metabolites, and one additional metabolite was detected. Metabolite **8** (**M8**) shows a reduction of the carboxylic acid moiety. However, limited by the poor MS/MS fragmentation of the aglycones and lack of NMR data, identification remains tentative. Further elucidation of the molecular structures was not possible. The only phase II products observed were glucuronidated metabolites of medicagenic acid (**M6**–**7**). Sulfation reactions were not observed, although they were predicted by Meteor and Biotransformer. To confirm that the in vitro model was capable to form sulfated conjugates, biotransformation of testosterone was examined as positive control. Testosterone was analyzed after hepatic biotransformation and proved the capability of the in vitro model to form both sulfated and glucuronidated conjugates. 

The phase II hydrophilic metabolites may be excreted via the kidneys and are often considered detoxifying as they usually possess lower activity compared to their parent aglycones. However, a number of glucuronide conjugates are known to be active. The 6-*O*-glucuronide of morphine, a widely used opioid analgesic, is the most well-known example of a glucuronidated metabolite possessing pharmacological activity greater than the parent aglycone [30]. Based on the observation of in vitro formation of glucuronidated hepatic metabolites, it is a possible hypothesis that these metabolites may be responsible for the urolithiatic activity [31]. A possible mode of action for prevention and dissolution of urinary stones could be the emulsifying properties of the glucuronidated metabolites, facilitating dissolution of crystals. 

## 3. Materials and Methods 

### 3.1. Chemicals

Ultrapure water with a resistivity of 18.2 MΩ·cm at 25 °C was generated with a Millipore™-purification system. Ultrahigh-Performance Liquid Chromatography (UHPLC)-grade MeOH, acetonitrile and formic acid were purchased from Biosolve (Dieuze, France), dichloromethane by Merck (Darmstadt, Germany). The following analytical standards were provided by Sigma–Aldrich (St. Louis, MO, USA): apigenin, benzoic acid, caffeic acid, +/− catechin, chlorogenic acid, cinnamic acid, coumarin, emodin, epicatechin, ferulic acid, isorhamnetin, naringenin, *p*-coumaric acid, protocatechuic acid, quercetin, quercitrin, rutin, salicylic acid, sinapic acid, β-sitosterol, stigmasterol, syringic acid, tannic acid, taxifolin, theophylline and vanillic acid. Luteolin and procyanidin B2 were provided by Santa Cruz Biotechnology (Santa Cruz, CA, USA). Gallic acid and *p*-hydroxybenzoic acid were provided by Carl Roth (Karlsruhe, Germany). Human liver S9 fraction and NADPH RS (Regenerating System) were purchased from XenoTech. Medicagenic acid was provided by Phytolab (Vestenbergsgreuth, Germany). All other chemicals and biochemicals were purchased from Sigma-Aldrich (St. Louis, MO, USA).

### 3.2. Preparation of Standard Solutions

Standard stock solutions of the analytical standards were prepared at a concentration of 1 mg mL^−1^ in UHPLC-grade MeOH for each analyte separately and stored in the dark at −80 °C. Dilutions of these solutions were prepared in 60:40 (*v*:*v*) MeOH:ammonium formate buffer (40 mM, aqueous). Standard stock and working solutions were stored at −80 °C in the dark.

### 3.3. Sample Preparation

Aerial parts from *H. hirsuta* were collected in d’Oujda, Morocco. A voucher specimen of the plant is kept at the Muséum National d’Histoire Naturelle—Institut Scientifique-Université Mohamed V Agdal, Morocco—(Reference number: 5902). The air-dried plant material of *H. hirsuta* was ground prior to extraction with a PF 10 basic Microfine grinder drive (IKA-Werke GmbH & Co. KG, Staufen, Germany) using a sieve mesh size 0.5 mm.

To cover the full range of constituents, a comprehensive extraction protocol was applied, previously described by Bijttebier et al. with minor adaptations [32,33]. Combination of extraction with H_2_O:EtOAc and CHCl_3_:MeOH:H_2_O afforded both polar and apolar compounds. Approximately 18 g of sample was subsequently wetted with 54 mL of water and 225 mL of EtOAc. The mixture was stirred for 2 h followed by 1 h of ultrasound-assisted extraction. The solvent was removed, and the residue was washed three times with 20 mL EtOAc. The extract was evaporated to dryness. CHCl_3_:MeOH:H_2_O extraction was performed by adding 18 g of sample to 180 mL CHCl_3_:MeOH:H_2_O (4:4:2). The mixture was stirred for 5 min, followed by 5 min of ultrasound-assisted extraction and stirred for 15 more min. The solvent was removed, and the residue was washed 3 times with 20 mL CHCl_3_:MeOH:H_2_O (4:4:2). The dried H_2_O:EtOAc extract was redissolved with the extract from the CHCl_3_:MeOH:H_2_O extraction method, leading to an apolar and a polar phase. Both phases were separated and dried.

### 3.4. Gastrointestinal Biotransformation

Many natural products are pro-drugs that are biotransformed and activated after oral administration. A previously developed and validated in vitro gastrointestinal biotransformation model was used to mimic human biotransformation processes [34]. This model allows the study of biotransformation processes in the stomach, small intestine and colon using a culture of pooled human feces, avoiding extensive in vivo studies.

The digestive juices and fecal suspension were formulated corresponding to the human conditions, previously described by Breynaert et al. and Peeters et al. [23,34]. Small adaptations to the method were made [35]. Briefly, the pepsin solution was prepared by dissolving 16% (*w*/*v*) of pepsin powder in 0.1 M HCl (622,000 FIP-U 100 mL^−1^). To obtain a pancreatin-bile mixture, 0.4% (*w*/*v*) pancreatin and 2.5% (*w*/*v*) bile were dissolved in 0.1 M NaHCO_3_ (32,000 FIP-U lipase, 143,600 FIP-U amylase, 16,400 FIP-U protease and 58.4 mmol bile L^−1^). A suspension of 10% (*w*/*v*) feces was prepared by homogenizing 3 human fecal samples with a sterile phosphate-buffer solution (0.1 M, pH 7.0). The phosphate-buffer solution consisted of NaH_2_PO_4_.2H_2_O (1.03% *w*/*v*), sodium thioglycolate broth (3.45% *v*/*v*) and glycerol (17%). Prior to use, the fecal suspension was cultivated. To 10% (*v*/*v*) pooled fecal suspension in phosphate buffer 90% (*v*/*v*) phosphate buffer was added in an anaerobic glove-box (5% H_2_, 5% CO_2_ and 90% N_2_) (Jacomex Glove Box T_3_, TCPS, Belgium). The bacteria were cultivated in an anaerobic environment for 1 h at 37 °C by continuously stirring. 

Three groups were included in the experiment: (1) samples containing the *Herniaria* extract, which were treated with digestive enzymes and fecal microflora (HEX); (2) negative control samples, also containing the extract with addition of digestive enzymes but not of fecal bacteria (NCHEX); and (3) method blanks, comprising an equal volume of solvent instead of extract and undergoing treatment with digestive enzymes and fecal bacteria (MB). For the samples, an amount of 300 mg lyophilized polar phase of the comprehensive extract of *H. hirsuta* was accurately weighed in triplicate. 

Gastrointestinal biotransformation was simulated in vitro. An aliquot of each sample was taken at several time points: before biotransformation (t_0_), after the gastric stage (S), after the small intestinal phase (SI) and at several time points during the colonic phase (after 2, 4, 6, 10, 14, 18, 22, 24, 32, 40, 48 and 72 h). Every sample was diluted with MeOH (1:2) and centrifuged for 5 min at 3500 rpm. The supernatant was collected, and samples were diluted 10 times with MeOH:H_2_O (60:40) before analysis.

### 3.5. Liver Biotransformation

Liver biotransformation mimicking phase I and II reactions was performed using medicagenic acid, the most abundant aglycone after gastrointestinal biotransformation. Hepatic biotransformation was simulated in vitro by using pooled S9 fractions, previously described by Van den Eede et al. [36]. Small adaptations to the method were made. Briefly, a mixture of 65 mM TRIS buffer (pH adjusted to 7.4 at 37 °C), human liver S9 fraction (1 mg mL^−1^ final protein concentration) and medicagenic acid (100 µM final concentration) was prepared in a total volume of 0.5 mL and preincubated in a shaking waterbath at 37 °C. The reaction was initiated by addition of an NADPH regenerating system (1 mM NADP, final concentration). Cofactors were added to expose the samples to phase II glucuronidation and sulfation: UDPGA (2 mM final concentration), GSH (2 mM final concentration) and PAPS (0.1 mM final concentration). The reaction was inhibited after 1 h by adding 0.5 mL of acetonitrile and storing the tubes on ice. Thereafter, the tubes were centrifuged for 5 min at 10,000 rpm (4 °C). The supernatant was collected and analyzed by LC–MS-MS. Blank samples were prepared as described above, replacing substrate by solvent. Negative control samples were prepared by immediately adding acetonitrile to quench the biotransformation reactions. A positive control was included by incubating testosterone (100 µM final concentration) [36].

### 3.6. Instrumental Analysis

For characterization of the *H. hirsuta* extract, an aliquot of 5 µL was injected with a CTC PAL™ autosampler (CTC Analytics) on a Waters Acquity UHPLC BEH SHIELD RP18 column (3.0 mm × 150 mm, 1.7 µm; Waters, Milford, MA, USA) and eluted with an Accela™ quaternary solvent manager. The temperature of the column was kept at 40 °C using a “Hot Pocket” column oven (Thermo Fisher Scientific, Waltham, MA, USA). The mobile phase solvents consisted of water + 0.1% formic acid (A) and acetonitrile + 0.1% formic acid (B). Compounds were eluted using the following gradient (min/B%): 0.00/0, 9.91/26, 18.51/65, 18.76/100, 20.76/100, 20.88/0, 23.00/0. For detection, an amMS (Q Exactive™; Thermo Fisher Scientific) was used with heated electrospray ionization (HESI). Spray voltage was set at −2.5 kV, sheath gas and auxiliary gas at 47 and 15 (adimensional), respectively, and capillary temperature at 350 °C. The PDA detector was set to scan from 190 to 800 nm. During the first analysis, full scan data were acquired in positive and negative ion mode over an *m*/*z* range of 120–1800 and resolving power was set at 70,000 at full width at half maximum (FWHM). Consecutively, a second analysis was performed to selectively fragment the generated ions by data-dependent fragmentation (ddMS^2^) in positive and negative ionization mode. Selective ion fragmentation was obtained by using a hybrid quadrupole-orbitrap MS analyzer (Q Exactive™, Thermo Fisher Scientific). The raw data were processed using XCalibur™ 3.0 software (Thermo Fisher Scientific).

For the qualitative UHPLC-UV-QTOF analyses of the biotransformation samples, an aliquot of 5 µL was injected on a Waters Acquity UHPLC BEH SHIELD RP18 column (3.0 mm × 150 mm, 1.7 µm; Waters). The temperature of the column was kept at 40 °C. The mobile phase solvents consisted of water + 0.1% formic acid (A) and acetonitrile + 0.1% formic acid (B), and the gradient was set as follows (min/B%): 0/2, 1/2, 14/26, 24/65, 26/100, 29/100, 31/2, 41/2. The flow rate was set at 0.4 mL min^−1^. For detection, accurate mass measurements were done using a Xevo G2-XS QTof spectrometer (Waters, Milford, MA, USA) coupled with an ACQUITY LC system equipped with MassLynx 4.1 software. During the first analysis, full scan data were recorded in ESI (+) and ESI (−) mode from *m*/*z* 50 to 2000, and the analyzer was used in sensitivity mode (approximate resolution: 22,000 FWHM). The spray voltage was set at either +1.5 kV and −1.0 kV; cone gas flow and desolvation gas flow at 50.0 L h^−1^ and 1000.0 L h^−1^ respectively; source temperature and desolvation temperature at 120 °C and 500 °C respectively. Data were also recorded using MS^E^ in positive and negative ionization modes, providing separate MS and MS^E^ data. A ramp collision energy from 20 V to 30 V was applied to obtain additional structural information. Leucine encephalin was used as lock mass. To monitor analytical drift and assess precision, quality control (QC) samples were injected after every time point. QC samples were prepared using a dilution of the standard solution mix (39 ng mL^−1^). 

### 3.7. Data Analysis

Dereplication was used as an untargeted workflow for rapid identification of the major compounds present in the extract, regardless of their chemical class. Global Natural Products Social Molecular Networking (GNPS, a web-based mass spectrometry open-access knowledge base) was used to facilitate identification [37]. Raw files were uploaded into the METABOLOMICS-SNETS-V2 workflow from GNPS (Global Natural Products Social Molecular Networking) in order to cluster the data based on their MS/MS fragmentation pattern. The precursor ion mass tolerance and the fragment ion mass tolerance were set at 2.0 Da and 0.5 Da respectively, and the cosine score was set at 0.7. The generated molecular network was visualized using Cytoscape 3.7.1 software.

To encounter the dynamic nature of the biotransformation process, a data analysis workflow for in vitro gastrointestinal biotransformation was implemented, previously developed and validated using hederacoside C as a model compound [23,27]. Briefly, data were converted to the open-source mzData format to allow further processing [38]. XCMS was used to convert the raw data into features via peak-picking, using following parameters: ppm = 10, peakwidth = c(5, 25, snthresh = 10, noise = 1000, mzdiff = 0.01, prefilter = c(3, 5000), integrate = 1. XCMS was followed by grouping, and EDGE was used for the extraction of significant differential profiles [39,40]. Tinderesting, an interactive Shiny app developed in R, was used to rate the quality of the resulting features. These ratings were used to train a random forest model for predicting experts response. The machine learning model provided a single score for each feature, referred to as tinderesting score, which allowed ranking of all features based on the difference over time between the three groups. The maximal score of 1 corresponds to the model labeling this feature as interesting, and the minimal score of 0 defines an uninteresting feature [23,27].

Several peaks in the output correspond to the same type of sample molecule, reflecting its isotope pattern. Isotopes were removed, using following parameters: IsoMass = 1.003355, IsoMassMaxDiff = 0.05, RTdiff = 10, corrThreshold = 0.8, ppmIsoDiffThreshold = 5.

The metabolic pathway of herniariasaponin H was studied since it is the most abundant saponin present in the extract and it is structurally related to hederacoside C, which was used for optimization of the workflow [23]. Both compounds contain a sapogenin linked to sugar moieties via the hydroxy groups at C-3 and C-28 of the aglycone. Herniariasaponin H comprises medicagenic acid as aglycone, which only differs from hederagenin by an additional hydroxy group at C-2 and a C-24 hydroxycarbonyl group instead of a hydroxymethyl group. However, the challenge in revealing the metabolic pathway of herniariasaponin H was that these experiments were not conducted with a single saponin, but with an herbal extract, comprising a complex mixture of compounds of high structural variety.

A suspect screening method, based on in silico metabolite prediction, was combined with a nontarget screening workflow to enhance the identification of products formed by in vitro liver biotransformation [41]. 

A list of potential biotransformation products was generated using Meteor Nexus 2.1 (Lhasa Limited, Leeds, UK). For phase I biotransformation, all redox and non-redox biotransformations were selected. For phase II biotransformation, *O*-glucuronidation, *O*-sulfonylation, acetylation and conjugation with amino acids were selected. The maximum depth was set at 3, and the maximum number of biotransformation products at 1000.

Biotransformer was used as an additional software tool to predict phase II metabolites. The tool uses both a knowledge-based approach and a machine-learning-based approach to predict biotransformation [42]. The SMILES string of medicagenic acid was uploaded, and “Phase I Transformation” and “Phase II Transformation” were selected separately. The generated csv file contained InChIKey, synonyms, major isotope mass, molecular formula, type of biotransformation reaction and precursor ID.

A nonarget screening workflow was conducted with MZmine 2.29 software (pluskal, mzmine 2) after converting the raw data files to the open-source mzXML format. Firstly, *m*/*z* features were detected using the centroid algorithm followed by a chromatogram building step. Resulting chromatograms were deconvoluted using the noise amplitude algorithm. Further data reduction occurred by deisotoping, keeping the lowest *m*/*z* value as the representative isotope. Meanwhile, chromatographic peaks were also filtered according to peak width: only peaks with a width between 0.05 and 1.00 min were retained. Next, peaks were aligned across samples using the random sample consensus (RANSAC) alignment algorithm correcting for nonlinear shifts in retention times. Finally, using the same rt and *m*/*z* range gap filler algorithm, any missing peaks were reiteratively extracted. The obtained *m*/*z* features were imported in R and processed as described by Vervliet et al. [43]. In brief, fold changes between samples and negative controls were calculated. A volcano plot was constructed to plot the *p*-value from a student t-test as a function of the calculated fold change for every feature. Features with a *p*-value lower than 0.05 and a log 10-fold change higher than 10 were selected for in depth investigation [41].

## 4. Conclusions

Since there is no prophylactic therapy for urinary stone disease, there is an urgent need for new drugs. An integrated strategy, based on natural prodrugs and their metabolites, is developed to characterize new lead compounds derived from *H. hirsuta*. The combination of a comprehensive extraction method and biotransformation via in vitro gastrointestinal and hepatic models, followed by metabolomics profiling, is an innovative concept to identify and further develop new lead compounds for drugs. 

Comprehensive extraction led to the tentative identification of 15 saponins that have not been reported before in *H. hirsuta*. The automated data analysis workflow used for unbiased screening for metabolites revealed the gastrointestinal biotransformation pathway of several compounds. A decrease in relative abundance over time was observed for the majority of all identified compounds, especially during the colon phase. Additionally, the relative abundance of saponin aglycones increased, illustrating the biotransformation of possible saponin prodrugs to their respective aglycones, which was also observed for flavonoids. Further hepatic biotransformation of the most abundant saponin aglycone revealed further biotransformation of medicagenic acid to different phase I and phase II metabolites, which can be used as a possible target for in vitro activity studies against urinary stones.

## Figures and Tables

**Table 1:** Chromatographic and Spectrometric Data of the Tentatively Identified Compounds in *H. hirsuta* Detected with a Generic LC-PDA-amMS Method for Moderately Polar Phytochemicals.

Com-Pound Number	Compound *^a^*	Molecular Formula	RT (min)		HESI Full MS *^b^*	HESI ddMS^2^	Neutral Losses	Max Abs. (nm)	Literature
1	quinic acid *^c^*	C_7_H_12_O_6_	1.54	neg	191.05611	-			
				pos	-	-			
2	gallic acid *^c^*	C_7_H_6_O_5_	4.06	neg	169.01412	-			
				pos	-	-			
3	protocatechuic acid [3,4-dihydroxybenzoic acid] *^c^*	C_7_H_6_O_4_	6.46	neg	153.01933	109.02950	109.02950: C_6_H_6_O_2_	259; 294	
				pos	-	-			
4	caffeyl hexose	C_15_H_18_O_9_	7.68	neg	341.08817	179.03439; 135.04439	179.03439: C_9_H_8_O_4_; 135.04439: C_9_H_8_O_4_ - CO_2_		
				pos	365.08380 [M+Na]^+^; 360.12839 [M+NH_4_]^+^	181.04962; 163.03906; 145.02861; 135.04443	181.04962: C_9_H_8_O_4_; 163.03906: C_9_H_8_O_4_ - H_2_O; 145.02861: C_9_H_6_O_4_ - 2H_2_O; 135.04443: C_8_H_6_O_2_		
5	asperulosidic acid	C_18_H_24_O_12_	8.01	neg	431.11975; 499.10757 [M-H+NaFA]^−^; 863.24756 [2M-H]^−^	329.09064; 161.04463	329.09064: C_14_H_18_O_9_; 161.04463: C_6_H_10_O_5_		
				pos	433.13428	271.08188; 187.06091; 145.04990; 127.03919	271.08188: C_18_H_24_O_12_ - C_6_H_10_O_5_; 187.06091: C_8_H_10_O_5_; 145.04990: C_6_H_8_O_4_; 127.03919: C_6_H_6_O_3_		
6	dihydrocaffeic acid *^c^*	C_9_H_10_O_4_	8.07	neg	181.05041	-			
				pos	-	-			
7	1-*O*-feruloylquinic acid *^d^*	C_17_H_20_O_9_	8.76	neg	367.10349; 435.09108 [M-H+NaFA]^−^	-			
				pos	369.11795	-			
8	chlorogenic acid [3.4-dihydroxycinnamoylquinic acid; 5-Caffeoylquinic acid] *^c^*	C_16_H_18_O_9_	8.94	neg	353.08685; 707.18355 [2M-H]^−^	191.05545	191.05611: C_7_H_12_O_6_	326	
				pos	355.10160; 377.08360 [M+Na]^+^; 731.17920 [2M+Na]^+^	163.03897	163.03897: C_9_H_6_O_3_		
9	isorhamnetin-3-*O*-rutinoside-7-*O*-glucoside *^d^*	C_34_H_42_O_21_	9.27	neg	785.21631; 853.20349 [M-H+NaFA]^−^	623.16479; 476.09396; 315.05124; 300.02750; 271.02466	623.16479: C_34_H_42_O_21_ - C_6_H_10_O_5_; 315.05124: C_16_H_12_O_7_; 300.02750: C_15_H_9_O_7_; 271.02466: C_14_H_7_O_6_		
				pos	787.22931	641.17114; 479.11838	641.17114: C_34_H_42_O_21_ - C_6_H_10_O_4_; 479.11838: C_34_H_42_O_21_ - C_6_H_10_O_4_ - C_6_H_10_O_5_		
10	chlorogenic acid *^c^*	C_16_H_18_O_9_	9.36	neg	353.08781	191.05611	191.05611: C_7_H_12_O_6_		
				pos	355.10236	163.03897			
11	Catechin *^c^*	C_15_H_14_O_6_	11.21	neg	289.07176	-		279	Mbark et al.
				pos	-	-			
12	epicatechin *^c^*	C_15_H_14_O_6_	11.25	neg	289.07176	-		280	
				pos	-	-			
13	procyanidin B2 *^c^*	C_30_H_26_O_12_	9.68	neg	577.13515	-			
				pos	579.14970	-			
14	caffeic acid *^c^*	C_9_H_8_O_4_	9.7	neg	179.03498	135.04515	135.04515: C_9_H_8_O_4_ - CO_2_		
				pos	181.04954	163.03915; 145.02861; 135.04443	163.03897: C_9_H_8_O_4_ - H_2_O		
15	1-*O*-feruloylquinic acid *^d^*	C_17_H_20_O_9_	10.51	neg	367.10358; 435.09106 [M-H+NaFA]^−^	193.05003; 173.04489; 134.03622	193.05003: C_10_H_10_O_4_; 173.04485: C_7_H_10_O_5_		
				pos	391.10001 [M+Na]^+^	145.02855; 149.05952; 117.03381; 177.05475; 194.05750	145.02855: C_9_H_4_O_2_; 149.05952: C_9_H_8_O_2_; 117.03381: C_8_H_4_O; 177.05475: C_10_H_10_O_4_ - H_2_O		
16	quercetin 3-rhamnosyl-(1->2)-rhamnosyl-(1->6)-glucoside or quercetin 3-rhamninoside	C_33_H_40_O_20_	11.05	neg	755.20471; 823.19204 [M-H+NaFA]^−^; 1511.41418 [2M-H]^−^	300.02771; 271.02499; 255.02994	300.02771: C_15_H_9_O_7_; 271.02499: C_14_H_8_O_6_; 255.02994: C_14_H_8_O_5_		
				pos	757.21765; 611.16085	465.1033; 303.05008	465.1033: C_33_H_40_O_20_ - 2C_6_H_10_O_4_; 303.05008: C_33_H_40_O_20_ - 2C_6_H_10_O_4_ - C_6_H_10_O_5_		
17	matairesinoside	C_26_H_32_O_11_	11.3	neg	565.19336 [M-H+FA]^−^	339.12378; 329.13812; 327.12405; 324.09967; 312.09885; 309.07953; 297.07669	339.12378: C_20_H_20_O_5_; 329.13812: C_19_H_22_O_5_; 327.12405: C_19_H_20_O_5_; 324.09967: C_19_H_17_O_5_; 312.09885: C_18_H_17_O_5_; 309.07953: C_18_H_14_O_5_; 297.07669: C_17_H_14_O_5_	355	
				pos	543.18420 [M+Na]^+^; 538.22786 [M+NH_4_]^+^	503.19199; 341.13672; 323.12738; 311.12756; 175.07526; 137.05983	503.19199: C_26_H_30_O_10_; 341.13672: C_20_H_20_O_5_; 323.12738: C_20_H_18_O_4_; 311.12756: C_19_H_18_O_4_; 175.07526: C_11_H_10_O_2_; 137.05983: C_8_H_8_O_2_		
18	isorhamnetin-*O*-glycoside (one hexosyl and two deoxyhexosyl units)	C_34_H_42_O_20_	11.74	neg	769.22040 [M-H]; 1539.44527 [2M-H]^−^; 632.32239	357.06104; 314.04349; 271.02512; 299.01981; 285.04065	632.32239: C_34_H_42_O_20_ - C_6_H_10_O_4_; 357.06104: C_18_H_14_O_8_; 314.04349: C_16_H_11_O_7_; 299.01981: C_15_H_8_O_7_; 285.04065: C_15_H_10_O_6_; 271.02512: C_14_H_8_O_6_	355	
				pos	771.23315; 625.17635; 479.11849	625.17743; 479.11874; 317.06540	625.17743: C_34_H_42_O_20_ - C_6_H_10_O_4_; 479.11874: C_34_H_42_O_20_ - 2C_6_H_10_O_4_; 317.06540: C_34_H_42_O_20_ - 2C_6_H_10_O_4_ - C_6_H_10_O_5_		
19	quercetin 3-*O*-β-(2″-*O*-α-L-rhamnopyranosyl)-glucopyranoside	C_27_H_28_O_17_	11.96	neg	623.12537; 1247.25657 [2M-H]^−^	301.03543	301.03543: C_27_H_28_O_17_ - C_6_H_10_O_4_ - C_6_H_8_O_6_	255; 355	van Dooren et al. (2016)
				pos	625.13943; 479.08180	479.08240; 303.04999	479.08180: C_27_H_28_O_17_ - C_6_H_10_O_4_; 303.04999: C_27_H_28_O_17_ - C_6_H_10_O_4_ - C_6_H_8_O_6_		
20	rutin *^c^*	C_27_H_30_O_16_	12.08	neg	609.14667; 677.13349 [M-H+NaFA]^−^	300.02762; 271.02481; 255.02990; 243.02990	300.02755: C_15_H_9_O_7_; 271.02481: C_14_H_8_O_6_; 255.02990: C_14_H_8_O_5_; 243.02990: C_13_H_8_O_5_	256; 355	van Dooren et al. (2016)
				pos	611.15973	465.10267; 303.04999	465.10275: C_21_H_22_O_12_; 303.04993: C_15_H_12_O_7_		
21	cynaroside *^c^*	C_21_H_20_O_11_	12.2	neg	447.09364	-			Wagner et al. (2013)
				pos	-	-			
22	hesperidin *^c^*	C_28_H_34_O_15_	12.24	neg	609.18153	-			
				pos	611.19705	-			
23	ferulic acid *^c^*	C_10_H_10_O_4_	12.34	neg	193.05063	178.02643	178.02643: C_9_H_7_O_4_		
				pos	195.06519	177.0547; 145.02855; 135.04424; 117.03378	177.0547: C_10_H_10_O_4_ - H_2_O; 149.05984: C_9_H_12_O_2_; 145.02855: C_9_H_4_O_2_; 135.04424: C_8_H_6_O_2_; 117.03378: C_8_H_4_O		
24	herniariasaponin 1	C_54_H_56_O_32_	12.59	neg	1215.2698	-			
				pos	1239.26343 [M+Na]^+^	-			
25	isoquercetin *^c^*	C_21_H_20_O_12_	12.61	neg	463.0882	300.02725; 271.02539; 255.02905; 243.02859; 151.00230	300.02725: C_15_H_9_O_7_; 271.02539: C_14_H_8_O_6_; 255.02905: C_14_H_8_O_5_; 243.02859: C_13_H_8_O_5_; 151.00230: C_7_H_4_O_4_		
				pos	465.10275	303.05008; 287.05533; 257.04483	303.05008: C_15_H_10_O_7_; 287.05533: C_15_H_10_O_6_; 257.04483: C_14_H_8_O_5_		
26	*p*-coumaric acid *^c^*	C_9_H_8_O_3_	12.62	neg	163.04007	119.05024	119.05024: C_8_H_8_O		
				pos	165.05462	147.04414	147.04414: C_9_H_6_O_2_		
27	2-(3.4-Dihydroxyphenyl)-5.7-dihydroxy-4-oxo-4*H*-chromen-3-yl-6-*O*-(2-*O*-acetyl-α-L-arabinopyranosyl)-β-D-glucopyranoside	C_28_H_30_O_17_	12.67	neg	637.14125; 1275.28809 [2M-H]^−^	463.09000; 300.02728	463.09000: C_21_H_20_O_12_; 300.02728: C_15_H_9_O_7_		
				pos	639.15543; 493.09749	465.10071; 303.04972	493.09749: C_28_H_30_O_17_ – C_6_H_10_O_4_; 465.10071: C_21_H_20_O_12_; 303.04972: C_15_H_10_O_7_		
28	miquelianin [quercetin-3-*O*-glucuronide]*^c^*	C_21_H_18_O_13_	12.89	neg	477.06746	301.03538; 271.02481 255.02990; 178.99860; 151.00368	301.03538: C_15_H_10_O_7_; 271.02481: C_14_H_8_O_6_; 255.02990: C_14_H_8_O_5_; 178.99860: C_8_H_4_O_5_; 151.00368: C_7_H_4_O_4_		
				pos	479.08202	303.04993; 257.04445	303.04993: C_15_H_10_O_7_; 257.04445: C_14_H_8_O_5_		
29	narcissin [isorhametin-3-*O*-rutinoside]	C_28_H_32_O_16_	12.89	neg	623.16211; 477.06749; 1247.33026 [2M-H]^−^	315.05072	477.06749: C_28_H_32_O_16_ - C_6_H_10_O_4_; 315.05072: C_28_H_32_O_16_ - C_6_H_10_O_4_ - C_6_H_10_O_5_	254; 355	van Dooren et al. (2016) Wagner et al. (2013)
				pos	625.14606	479.11804; 317.06586	479.11804: C_28_H_32_O_16_ - C_6_H_10_O_4_; 317.06586: C_28_H_32_O_16_ - C_6_H_10_O_4_ - C_6_H_10_O_5_		
30	avicularin [quercetin-3-*O*-arabinoside] *^c^*	C_20_H_18_O_11_	13.16	neg	433.0782	301.03586		256; 355	
				pos	435.09219	-			
31	apigetrin *^c^*	C_21_H_24_O_9_	13.27	neg	431.09837	268.03827	268.03827: C_15_H_9_O_5_		
				pos	433.11292	-			
32	astragalin [kaempferol 3-*O*-glucoside] *^c^*	C_21_H_20_O_11_	13.34	neg	447.09381	284.03314; 255.03024; 227.03455	284.03314: C_15_H_9_O_6_; 255.03024: C_14_H_8_O_5_; 227.03455: C_13_H_8_O_4_	265; 348	
				pos	449.10814	-			
33	quercitrin *^c^*	C_21_H_20_O_11_	13.35	neg	447.09328	-		265; 346	
				pos	449.10784	-			
34	salicylic acid *^c^*	C_7_H_6_O_3_	14.07	neg	137.02442	93.03459	93.03459: C_6_H_6_O	236; 302	
				pos	-	-			
35	herniariasaponin F	C_59_H_94_O_28_	14.17	neg	1249.58482; 647.29342	501.32303; 439.32358	647.29342: C_30_H_46_O_6_ + C_6_H_10_O_4_; 501.32303: C_30_H_46_O_6_; 439.32358: C_30_H_46_O_6_ - H_2_O - CO_2_		Mbark et al. (2000)
				pos	1273.58240 [M+Na]^+^; 1268.62600 [M+NH_4_]^+^; 1122.56885; 645.27747; 457.33133	771.25336	457.33133: C_29_H_44_O_4_; 771.25336: 2C_6_H_10_O_5_ + 2C_6_H_10_O_4_ + C_5_H_8_O_4_ + Na		
36	acetylated medicagenic acid + two hexosyl + two deoxyhexosyl + one pentosyl moieties *^e^*	C_61_H_96_O_29_	14.53	neg	1291.59644	1249.59241; 1231.57312; 821.43506; 543.33441; 501.32294; 483.31107; 439.32251	821.43506: C_61_H_96_O_29_ - 2C_6_H_10_O_5_ - C_6_H_10_O_4_; 543.33441: C_32_H_48_O_7_; 501.32294: C_30_H_46_O_6_; 483.31107: C_30_H_46_O_6_ - H_2_O; 439.32251: C_30_H_46_O_6_ - H_2_O - CO_2_		
				pos	1310.63818 [M+NH_4_]^+^	-			
37	acetylated medicagenic acid + three hexosyl + one deoxyhexosyl moieties *^e^*	C_56_H_88_O_26_	14.61	neg	1175.549	705.38928; 543.33337; 501.32281; 439.32205	705.38928: C_32_H_48_O_7_ + C_6_H_10_O_5_; 543.33337: C_32_H_48_O_7_; 501.32281: C_30_H_46_O_6_; 439.32205: C_30_H_46_O_6_ - H_2_O - CO_2_		Schröder et al. (1993)
				pos	1194.59088 [M+NH_4_]^+^	-			
38	medicagenic acid + two hexosyl + two deoxyhexosyl + two pentosyl + 1 glucuronic acid moiety *^e^*	C_70_H_110_O_38_	14.89	neg	1557.6595; 1411.60317	1235.56738; 983.57922; 796.23248; 439.32043	1411.60317: C_70_H_110_O_38_ - C_6_H_10_O_4_; 1235.56738: C_70_H_110_O_38_ - C_6_H_10_O_4_ - C_6_H_8_O_6_; 439.32043: C_29_H_44_O_3_		Mbark et al. (2000)
				pos	1581.65795 [M+Na]^+^	1259.56189; 741.24219; 595.18250; 445.13116			
39	zanhic acid + one hexose + four deoxyhexoses + two pentoses + one glucuronic acid moiety *^e^*	C_76_H_120_O_42_	14.92	neg	1703.71708; 851.35632 [M-2H]^2−^	1381.62830; 823.41089; 517.31879; 455.31766; 839.40845	1381.62830: C_76_H_120_O_42_ - C_6_H_10_O_4_ - C_6_H_8_O_6_; 517.31879: C_30_H_46_O_7_; 455.31766: C_30_H_46_O_7_ - H_2_O - CO_2_; 839.40845: C_30_H_47_O_7_ + C_6_H_10_O_4_ + C_6_H_8_O_6_		
				pos	1727.71399 [M+Na]^+^; 1722.75891 [M+NH_4_]^+^	-			
40	herniariasaponin E	C_55_H_86_O_25_	15.03	neg	1145.5387; 1213.52771 [M-H+NaFA]^−^; 572.26715; 595.26941 [M-2H-FA]^2−^	543.33105; 483.31180; 439.32227; 601.19806; 439.32208	543.33105: C_32_H_48_O_7_; 483.31180: C_30_H_46_O_6_ - H_2_O; 439.32227: C_30_H_46_O_6_ - H_2_O - CO_2_; 601.19806: C_5_H_8_O_4_ + C_6_H_10_O_4_ + 2C_6_H_10_O_5_; 439.32208: C_5_H_8_O_4_ + C_6_H_10_O_4_ + C_6_H_10_O_5_		Mbark et al. (2000)
				pos	1169.53381 [M+Na]^+^; 1164.57869 [M+NH_4_]^+^	545.34430	545.34430: C_32_H_48_O_7_		
41	acetylated medicagenic acid + three hexosyl moieties *^e^*	C_50_H_78_O_22_	15.06	neg	1029.49191; 1075.49754 [M-H+FA]^−^	543.33105; 483.31180; 439.32227; 791.07910; 585.34424	543.33105: C_32_H_48_O_7_; 483.31180: C_30_H_44_O_5_; 439.32227; 791.07910: C_29_H_28_O_26_		
				pos	1048.53173 [M+NH_4_]^+^; 1053.48612 [M+Na]^+^	-			
				pos	1398.65376 [M+NH_4_]^+^	-			
42	medicagenic acid + three deoxyhexosyl + one pentosyl + one glucuronic acid moiety *^e^*	C_59_H_92_O_28_	15.12	neg	1247.569	925.48315; 501.31976			
				pos	1271.56687 [M+Na]^+^	1127.49792; 949.47644; 701.34851; 525.32062; 447.14737			
43	zanhic acid + three deoxyhexosyl + one pentosyl + one glucuronic acid moiety *^e^*	C_59_H_92_O_29_	15.24	neg	1263.5657	1163.15430; 1081.94128			
				pos	1282.60583 [M+NH_4_]^+^	-			
44	medicagenic acid + two hexosyl + three deoxyhexosyl + two pentosyl + one glucuronic acid moiety *^e^*	C_76_H_120_O_42_	15.33	neg	1703.7174	1381.62830; 1219.22852; 879.30701; 843.52264; 823.41089; 501.32275; 439.31934			
				pos	1727.71399 [M+Na]^+^; 1722.75891 [M+NH_4]*_^+^	-			
45	medicagenic acid + one hexosyl + four deoxyhexosyl + two pentoses + one glucuronic acid moiety *^e^*	C_76_H_120_O_41_	15.52	neg	1687.7229	1365.63477; 998.50537; 823.42078			
				pos	1711.71936 [M+Na]^+^	-			
46	medicagenic acid + three deoxyhexosyl + two pentosyl + one glucuronic acid moiety *^d,e^*	C_64_H_100_O_32_	15.12; 15.62	neg	1379.6127	1057.52454; 925.48077; 823.41821; 501.32251; 439.32050	1057.52454: C_64_H_100_O_32_ - C_6_H_8_O_6_ - C_6_H_10_O_4_; 925.48077: C_64_H_100_O_32_ - C_6_H_8_O_6_ - C_6_H_10_O_4_ – C_5_H_8_O_4_; 823.41821: C_35_H_68_O_21_; 501.32251: C_30_H_46_O_6_; 439.32050: C_30_H_46_O_6_ - H_2_O - CO_2_		
				pos	1403.60791 [M+Na]^+^	1259.54272; 1081.51782; 847.40808; 579.18964			
47	medicagenic acid + one hexosyl + two deoxyhexosyl + two pentosyl + one glucuronic acid moiety *^e^*	C_64_H_100_O_33_	15.67	neg	1395.6069	439.32248; 1219.58301	439.32248: C_30_H_46_O_6_ - H_2_O - CO_2_; 1219.58301: C_64_H_100_O_33_ - C_6_H_8_O_6_		Charrouf et al. (1995)
				pos	1419.60059 [M+Na]^+^	-			
48	herniariasaponin B	C_65_H_102_O_33_	15.83	neg	1409.6244	1087.53479; 823.41272; 501.32300; 439.32343	1087.53479: C_65_H_102_O_33_ - C_6_H_10_O_4_ - C_6_H_8_O_6_; 823.41272; 501.32300: C_30_H_46_O_6_; 439.32343: C_30_H_46_O_6_ - H_2_O - CO_2_		Charrouf et al. (1995)
				pos	1433.61982 [M+Na]^+^; 1428.66283 [M+NH_4_]^+^; 1411.63638	-			
49	acetylated medicagenic acid + one deoxyhexosyl + two pentosyl moieties *^e^*	C_47_H_74_O_19_	16	neg	941.47546	501.32312; 439.32037; 821.43097; 543.33167	821.43097: C_47_H_74_O_19_ - C_5_H_8_O_4_; 543.33167: C_32_H_48_O_7_; 501.32312: C_30_H_46_O_6_; 439.32037: C_30_H_46_O_6_ - H_2_O - CO_2_		
				pos	960.51593 [M+NH_4_]^+^; 965.47021 [M+Na]^+^	463.14227: C_17_H_27_O_13_Na			
50	quercetin *^c^*	C_15_H_10_O_7_	16.4	neg	301.03538	271.02539; 178.99774; 151.00273; 121.02832	271.02539: C_14_H_8_O_6_; 178.99774: C_8_H_4_O_5_; 151.00273: C_7_H_4_O_4_; 121.02832: C_7_H_6_O_2_	255; 372	
				pos	303.04993	285.03964; 257.04440; 229.04947; 201.05476; 165.01811; 153.01846; 137.02357	285.03964: C_15_H_8_O_6_; 257.04440: C_14_H_8_O_5_; 229.04947: C_13_H_8_O_4_; 201.05476: C_12_H_8_O_3_; 165.01811: C_8_H_4_O_4_; 153.01846: C_7_H_4_O_4_; 137.02357: C_7_H_4_O_3_		
51	acetylated medicagenic acid + one hexosyl + two deoxyhexosyl + one pentosyl + one glucuronic acid moiety*^e^*	C_61_H_94_O_30_	16.76	neg	652.28625 [M-2H]^2−^; 1305.57764	439.32321; 585.20691	439.32321: C_29_H_43_O_3_; 585.20691: C_5_H_8_O_4_ + 2C_6_H_10_O_4_ + C_6_H_10_O_5_		
				pos	1329.57288 [M+Na]^+^; 1324.61719 [M+NH_4_]^+^	-			
52	naringenin *^c^*	C_15_H_12_O_5_	16.8	neg	271.06120	253.05069	253.05069: C_15_H_10_O_4_		
				pos	273.07575	-			
53	apigenin *^c^*	C_15_H_10_O_5_	17.73	neg	269.04555	225.05510; 151.00255; 117.03339	225.05510: C_14_H_10_O_3_; 151.00255: C_7_H_4_O_4_		
				pos	271.06010	153.01845	153.01845: C_7_H_4_O_4_		
54	isorhamnetin *^c^*	C_16_H_12_O_7_	17.87	neg	315.05103	300.02762; 271.02399; 243.02707; 216.04128; 164.01071; 151.00258	300.02762: C_15_H_9_O_7_		
				pos	317.06555	302.04205; 285.03882; 153.01846	153.01846: C_7_H_4_O_4_		
55	luteolin *^c^*	C_15_H_10_O_6_	18.09	neg	285.04046	199.03967; 151.00212; 133.02821	199.03967: C_12_H_8_O_3_; 151.00212: C_7_H_4_O_4_; 133.02821: C_8_H_6_O_2_		
				pos	287.05501	153.01787; 135.04414	153.01787: C_7_H_4_O_4_; 135.04414: C_8_H_6_O_2_		
56	kaempferol *^c^*	C_15_H_10_O_6_	18.09	neg	285.04046	-		265; 364	
				pos	287.05501	258.05115; 231.06519; 165.01807; 153.01816; 121.02866	258.05115: C_14_H_9_O_5_; 231.06519: C_13_H_10_O_4_; 165.01807: C_8_H_4_O_4_; 153.01816: C_7_H_4_O_4_; 121.02866: C_7_H_4_O_2_		
57	miquelianin [quercetin 3-*O*-glucuronide] *^c^*	C_21_H_18_O_13_	12.89–13.49	neg	477.0675	301.03552; 271.02505; 255.03020;178.99788; 151.00258		257; 357	
				pos	479.0824	303.04993; 257.04370			
58	herniariasaponin G	C_53_H_84_O_24_	14.61	neg	1103.52829; 574.26304 [M-2H+FA]^−^	821.43842; 501.32306; 439.32156	821.43842: C_36_H_70_O_20_; 501.32306: C_30_H_46_O_6_; 439.32156: C_30_H_46_O_6_ -H_2_O - CO_2_		van Dooren et al. (2016)
				pos	1127.52344 [M+Na]^+^; 1122.56841 [M+NH_4_]^+^; 811.44629; 503.33636; 457.33087	973.50128; 811.45264; 649.39624; 503.33704; 457.33188	811.44629: C_53_H_84_O_24_ - C_5_H_8_O_4_ - C_6_H_10_O_5_; 503.33636: C_30_H_46_O_6_; 973.50128: C_53_H_84_O_24_ - C_5_H_8_O_4_; 649.39624: C_53_H_84_O_24_ - C_5_H_8_O_4_ - 2C_6_H_10_O_5_		
59	medicagenic acid + two hexosyl + two deoxyhexosyl + two pentosyl + one glucuronic acid moiety *^e^*	C_70_H_110_O_38_	14.89–15.38	neg	1557.6595; 1411.60317	1235.56738; 983.57922; 796.23248; 439.32043	1411.60317: C_70_H_110_O_38_ - C_6_H_10_O_4_		
				pos	1581.65795 [M+Na]^+^	1259.56189; 741.24219; 595.18250; 445.13116			
60	medicagenic acid + one hexosyl + two deoxyhexosyl + two pentosyl + one glucuronic acid moiety *^e^*	C_64_H_100_O_33_	14.92–15.67	neg	1395.6069	1219.583			
				pos	-	-			
61	herniariasaponin H *^d^*	C_70_H_110_O_37_	15.12; 15.66; 15.72	neg	1541.6657	1446.38293; 1219.58069; 823.41711; 501.32587	1219.58069: C_70_H_110_O_37_ - C_5_H_8_O_4_ - C_6_H_8_O_6_; 823.41711: C_70_H_110_O_37_ - C_6_H_10_O_5_ - 2C_5_H_8_O_4_ - 2C_6_H_10_O_4_; 501.32587: C_30_H_46_O_6_		van Dooren et al. (2016)
				pos	1565.66118 [M+Na]^+^; 1560.70778 [M+NH_4_]^+^	1243.57349; 905.31189; 741.24255			
62	chlorogenic acid *^d^*	C_16_H_18_O_9_	7.31; 8.88	neg	353.08685	191.05544; 179.03427; 173.04494	191.05544: C_7_H_12_O_6_; 179.03427: C_9_H_8_O_4_; 173.04494: C_7_H_12_O_6_ - H_2_O		
				pos	355.1016; 377.08360 [M+Na]^+^	163.03915; 145.02861; 135.04443	163.03915: C_9_H_8_O_4_ - H_2_O; 145.02861: C_9_H_8_O_4_ - 2H_2_O; 135.04443: C_8_H_8_O_2_		
63	1-*O*-feruloylquinic acid *^d^*	C_17_H_20_O_9_	9.20–10.62	neg	367.10349; 435.09108 [M-H+NaFA]^−^	193.05003; 173.04485; 134.03622			Jaiswal et al. (2011)
				pos	369.11748; 391.09949 [M+Na]^+^	145.02855; 149.05952; 117.03381; 177.05475; 194.05750			

*^a^* If not specified otherwise: tentative identification based on accurate mass *^b^* Deprotonated and protonated molecules in negative and positive mode, respectively, unless stated otherwise *^c^* Identification with an analytical standard *^d^* Tentatively identified compound or an isomer *^e^* Tentative identification of saponins that have never been reported before in *H. hirsuta*.

**Table 2:** Most probable sum formula and ppm deviation for the fragment ions of *m*/*z* 1703.71734.

*m*/*z* [M-H]^−^	Sum Formula	∆ ppm
1703.71838	C_76_H_120_O_42_	0.141
1381.62830	C_64_H_102_O_32_	0.113
879.30701	C_34_H_56_O_26_	9.445
823.41089	C_42_H_64_O_16_	−1.541
501.32275	C_30_H_45_O_6_	1.172
439.31934	C_29_H_44_O_3_	−5.528
